# Higher Sensitivity and Reproducibility of Wavelet-Based Amplitude of Resting-State fMRI

**DOI:** 10.3389/fnins.2020.00224

**Published:** 2020-03-31

**Authors:** Fei-Fei Luo, Jian-Bao Wang, Li-Xia Yuan, Zhi-Wei Zhou, Hui Xu, Shao-Hui Ma, Yu-Feng Zang, Ming Zhang

**Affiliations:** ^1^The Key Laboratory of Biomedical Information Engineering of Ministry of Education, Institute of Biomedical Engineering, School of Life Sciences and Technology, Xi’an Jiaotong University, Xi’an, China; ^2^Department of Medical Imaging, The First Affiliated Hospital, Xi’an Jiaotong University, Xi’an, China; ^3^Institutes of Psychological Sciences, Hangzhou Normal University, Hangzhou, China; ^4^Zhejiang Key Laboratory for Research in Assessment of Cognitive Impairments, Hangzhou, China; ^5^Center for Cognition and Brain Disorders, The Affiliated Hospital, Hangzhou Normal University, Hangzhou, China

**Keywords:** amplitude of low-frequency fluctuation, wavelet transform, resting-state fMRI, sensitivity, reproducibility

## Abstract

The fast Fourier transform (FFT) is a widely used algorithm used to depict the amplitude of low-frequency fluctuation (ALFF) of resting-state functional magnetic resonance imaging (RS-fMRI). Wavelet transform (WT) is more effective in representing the complex waveform due to its adaptivity to non-stationary or local features of data and many varieties of wavelet functions with different shapes being available. However, there is a paucity of RS-fMRI studies that systematically compare between the results of FFT versus WT. The present study employed five cohorts of datasets and compared the sensitivity and reproducibility of FFT-ALFF with those of Wavelet-ALFF based on five mother wavelets (namely, db2, bior4.4, morl, meyr, and sym3). In addition to the conventional frequency band of 0.0117–0.0781 Hz, a comparison was performed in sub-bands, namely, Slow-6 (0–0.0117 Hz), Slow-5 (0.0117–0.0273 Hz), Slow-4 (0.0273–0.0742 Hz), Slow-3 (0.0742–0.1992 Hz), and Slow-2 (0.1992–0.25 Hz). The results indicated that the Wavelet-ALFF of all five mother wavelets was generally more sensitive and reproducible than FFT-ALFF in all frequency bands. Specifically, in the higher frequency band Slow-2 (0.1992–0.25 Hz), the mean sensitivity of db2-ALFF results was 1.54 times that of FFT-ALFF, and the reproducibility of db2-ALFF results was 2.95 times that of FFT-ALFF. The findings suggest that wavelet-ALFF can replace FFT-ALFF, especially in the higher frequency band. Future studies should test more mother wavelets on other RS-fMRI metrics and multiple datasets.

## Introduction

Using resting-state functional magnetic resonance imaging (RS-fMRI), Biswal et al. (1995) observed for the first time that the low-frequency (<0.1 Hz) fluctuation (LFF) was highly correlated between sensorimotor cortices. Subsequently, RS-fMRI has attracted significant attraction. While vast majority of RS-fMRI studies investigated brain networks by analyzing the relationship between different brain areas, a few studies focused on the local brain activity.

The amplitude of LFF (ALFF) is the simplest metric to measure the local spontaneous activity of every single time series. While most RS-fMRI studies used a conventional frequency band of 0.01–0.08 Hz, sub-bands were also extensively used in research on brain disorders (Liu et al., 2014; Giménez et al., 2017; Li et al., 2017a, b; Wang et al., 2018) following the study by Zuo et al. (2010) in which a few sub-bands were mentioned including Slow-5 (0.01–0.027 Hz), Slow-4 (0.027–0.073 Hz), Slow-3 (0.073–0.198 Hz), and Slow-2 (0.198–0.25 Hz). Although the very low frequency band < 0.01 Hz (Slow-6) was not included in most studies, a few studies indicated that Slow-6 was meaningful either physiologically (Lv et al., 2013; Zhang et al., 2015) or pathophysiologically (Wang et al., 2015). Therefore, frequency-dependent analysis should be included in studies in addition to the conventional frequency band.

The ALFF is calculated with a fast Fourier transform (FFT) (Zang et al., 2007), wherein the energy of a time series is decomposed by Fourier transform (FT) into a set of sinusoidal functions at different frequencies. However, the fMRI signals are complex waveforms, and thus, it is difficult for a set of stationary sinusoidal functions to detect transient phenomena, such as spikes (Bullmore et al., 2004; Mallat, 2009). Patel et al. (2014) mentioned “wavelet analysis offers a powerful set of tools for analyzing the properties of complex time series (Daubechies and Heil, 1992; Mallat, 2009).” The natural adaptivity of the wavelet transform (WT) to local or non-stationary features of data and many varieties of wavelet functions with different shapes being available makes it more effective in depicting complex waveform than the FFT (Bullmore et al., 2004; Zhang et al., 2014).

Currently, many RS-fMRI studies use WT. Most existing WT RS-fMRI studies use WT for functional connectivity analysis (Achard et al., 2006; Deshpande et al., 2010; Spoormaker et al., 2010; Schröter et al., 2012; Yaesoubi et al., 2017; Váša et al., 2018). In functional connectivity analysis domain, some studies have analyzed the transformed wavelet coefficients (Achard et al., 2006), whereas others have analyzed the transformed wavelet-filtered time series (Xu et al., 2016) of certain frequency ranges. As previously mentioned, functional connectivity describes the relationship between different brain areas. ALFF describes the activity of a single time series, and it is widely used for precisely localizing abnormal brain activity. Another similar metric is the power of the time series. It should be noted that the power is proportional to the square of ALFF (Zang et al., 2007). To the best of the authors’ knowledge, all ALFF studies are based on FFT, and only two studies applied WT to analyze the power of a single time series (Salomon et al., 2011; Bajaj et al., 2013). Fox example, a study compared WT-based power between two RS-fMRI conditions, i.e., acute tryptophan depletion diet versus control diet (Salomon et al., 2011). Although theoretically WT is more valid in depicting complex time series than FFT, there is a paucity of studies comparing the WT-based ALFF (Wavelet-ALFF) with the FFT-ALFF. Moreover, there are several mother wavelet bases. However, two existing WT-based power studies did not compare different wavelets.

In the present study, we applied five mother wavelets, namely, Daubechies 2 (db2) (Bullmore et al., 2004; Salomon et al., 2011; Zhang et al., 2016), biorthogonal 4.4 (bior4.4) (Laine, 2000; Van De Ville et al., 2003; Mutihac, 2006), Morlet (morl) (Chang and Glover, 2010; Bajaj et al., 2013; Omidvarnia et al., 2017; Yaesoubi et al., 2017), Meyer (meyr) (Behjat et al., 2015), and Symlets 3 (sym3) (Khullar et al., 2011) to calculate ALFF and compared the sensitivity and reproducibility between Wavelet-ALFF and FFT-ALFF in multiple frequency bands and multiple cohorts to explore whether Wavelet-ALFF is superior to FFT-ALFF and as to which mother wavelet is more superior.

## Materials and Methods

### Subjects and Data Acquisition

In this study, we used two MRI datasets, namely, EOEC and ADHD-200. The reason for using the two datasets is explained in Section “Discussion.” All data acquisitions were approved by the corresponding institutional ethics committees. All subjects provided the informed consent before data collection. Additionally, all subjects did not have a history of neurological disease or psychiatric disorders.

The EOEC dataset consisted of 31 right-handed healthy subjects (21.8 ± 1.8 years old, 15 females). The subjects experienced two 8-min RS-fMRI sessions, namely, one with eyes open (EO) and the other with eyes closed (EC). The order of the two sessions was counterbalanced across subjects. Specifically, MRI data were collected by a GE MR750 3T scanner (GE Healthcare, Milwaukee, WI, United States) with an eight-channel head receiving coil. During data collection, foam cushions were applied to reduce head movement, and earplugs were applied to diminish scanner noise. The scanning parameters of RS-fMRI data were as follows: TR/TE = 2000/30 ms, flip angle = 60°, 43 slices, thickness/gap = 3.4/0 mm, and FOV = 220 mm × 220 mm with an in-plane resolution of 3.44 mm × 3.44 mm. The resting-state BOLD scan lasted for 8 min and produced 240 images. A three-dimensional (3D) T1 was obtained with a spoiled gradient-recalled pulse sequence with the following parameters: TR/TE = 8.1/3.1 ms, flip angle = 9°, 176 sagittal slices, thickness = 1 mm, FOV = 250 mm × 250 mm.

The ADHD-200 dataset was from the publicly available dataset “The ADHD-200 Consortium”.^1^ The ADHD-200 dataset contains the RS-fMRI and anatomical MRI data of children with attention deficit hyperactivity disorder (ADHD) and typically developing children (TDC). The ADHD-200 dataset was provided by eight independent imaging sites and was divided into the training and test sets by the ADHD-200 Global Competition. The current study only used the data from four imaging sites, i.e., NYU, PKU1, PKU2, and PKU3 as described in a previous study (Wang et al., 2017). Given that the PKU3 only has male subjects, the female subjects in NYU, PKU1, and PKU2 were not analyzed. Additionally, the current study also removed the data from left-handed subjects. Finally, the data from 58 subjects in NYU, 30 subjects in PKU1, 56 subjects in PKU2, and 38 subjects in PKU3 were used after age matching between children with ADHD and TDC (Wang et al., 2017).

### Data Preprocessing

All the images were preprocessed *via* a MATLAB toolbox, Data Processing Assistant for RS-fMRI (DPARSF) (Yan and Zang, 2010) which was based on Statistical Parametric Mapping (SPM8)^2^ and RS-fMRI Data Processing Toolkit (REST) (Song et al., 2011). The first 10 time points (20 s) were discarded because the subject took time to adapt to the scanning noise and additionally for the scanner to calibrate (Zang et al., 2004; Wang et al., 2017; Yan et al., 2019). Given that there were only 170 time points in NYU data, we retained 170 time points for PKU1, PKU2, and PKU3.

The preprocessing steps included slice time correction, head motion correction, spatial normalization (resampled into 3 mm × 3 mm × 3 mm), spatial smoothing (6-mm isotropic Gaussian kernel), and nuisance covariates regression (head motion effect using Friston 24-parameter model, white matter, and cerebrospinal fluid signal).

### Fast Fourier Transform–Amplitude of Low-Frequency Fluctuation Calculation

The FFT-ALFF was calculated by using the DPARSF software (Yan and Zang, 2010). As described in a previous study, the averaged square root of power spectrum obtained with the FFT across the given frequency band was considered as FFT-ALFF (Zang et al., 2007; Zuo et al., 2010). The FFT-ALFF was standardized by dividing each voxel’s FFT-ALFF by the “global” mean FFT-ALFF (Zang et al., 2007). It should be noted that “global” denotes a group mask. Some small parts of the brain were not covered during scanning for some subjects in the ADHD-200 dataset, and thus, we created a group mask in a manner similar to a previous study (Wang et al., 2017) in which the brain area of more than 80% of subjects were covered. The current study calculated FFT-ALFF in the conventional band (0.0117–0.0781 Hz) and five sub-bands as previously defined. These are the Slow-6 (0–0.0117 Hz), Slow-5 (0.0117– 0.0273 Hz), Slow-4 (0.0273–0.0742 Hz), Slow-3 (0.0742–0.1992 Hz), and Slow-2 (0.1992–0.25 Hz) (Zuo et al., 2010; Wang et al., 2015, 2017; Zhang et al., 2015).

### Wavelet–Amplitude of Low-Frequency Fluctuation Calculation

In this study, the continuous WT (CWT) was implemented *via* MATLAB 2014a Wavelet Toolbox (MathWorks, Natick, MA, United States). The CWT wavelet coefficient is defined as the convolution of the time series *x*(*t*) with the scaled and translated version of a mother wavelet function ψ_*k*,*s*_(*t*) (Torrence and Compo, 1998) as shown below:

(1) $$C\,W\,T\,\left(k,s\right)=\frac{1}{\sqrt{s}}\cdot \int_{-\infty }^{+\infty }x\,\left(t\right)\cdot \psi^{*}\,\left(\frac{t-k}{s}\right)\,d\,t$$

where *x*(*t*) denotes the time series, ψ_*k*,*s*_(*t*) denotes the mother wavelet function, *s* denotes wavelet scale (64 frequency bins in the current study, between 0 and 0.25 Hz at an interval of 0.0039 Hz), *k* denotes the localized time index (*k* ∈ [1,170] and [1,230] for EOEC dataset and ADHD-200 dataset, respectively), and ^∗^ denotes the complex conjugate (Poza et al., 2014; Morabito et al., 2017).

We used five mother wavelets which have been used in previous fMRI literature, including db2 (Bullmore et al., 2004; Salomon et al., 2011; Zhang et al., 2016), bior4.4 (Laine, 2000; Van De Ville et al., 2003; Mutihac, 2006), morl (Chang and Glover, 2010; Bajaj et al., 2013; Omidvarnia et al., 2017; Yaesoubi et al., 2017), meyr (Behjat et al., 2015), and sym3 (Khullar et al., 2011). The traces for the five wavelets are shown in Supplementary Figures S1–S5, respectively.

As mentioned in a previous study, “a relatively high value of the coefficient is given in the product with the wavelet if there exists a spectral component of the signal corresponding to the value of *s* at a location *k*” (Morabito et al., 2017). Wavelet-ALFF was calculated by first adding up the wavelet coefficients at all time points for each frequency point, and the averaged coefficient across a given frequency band was then obtained as defined below:

(2) $$W\,a\,v\,e\,l\,e\,t\,\text{-}\,A\,L\,F\,F=\frac{1}{m}\,\sum_{i=1}^{n}\left|C\,W\,T_{i,j}\right|,j=s_{1}\,\ldots \,s_{m}$$

where |*CWT*_*i*,*j*_| denotes the absolute value of wavelet coefficient at time point *i* at a given frequency point *j*; *n* denotes the total amount of wavelet coefficient at a given frequency point; and *m* denotes the total number of frequency points across a given frequency band. In the current study, we calculated the Wavelet-ALFF in the conventional frequency band of 0.0117–0.0781 Hz and five sub-bands, i.e., Slow-6 (0–0.0117 Hz), Slow-5 (0.0117–0.0273 Hz), Slow-4 (0.0273–0.0742 Hz), Slow-3 (0.0742–0.1992 Hz), and Slow-2 (0.1992–0.25 Hz). For standardization purpose as did for FFT-ALFF, each voxel’s Wavelet-ALFF was divided by the “global” (i.e., a group mask as described in Section “Fast Fourier Transform–Amplitude of Low-Frequency Fluctuation Calculation” mean Wavelet-ALFF.

### t-Tests on Amplitude of Low-Frequency Fluctuation Maps of Each Frequency Band

Paired *t*-tests between EO and EC conditions were performed for the EOEC dataset. Two-sample *t*-tests between ADHD group and TDC group were performed in each cohort for ADHD-200 dataset. As Jia et al. (2018) have recently reported, stringent or liberal multiple comparison correction could not control the false discoveries across multiple studies when the effect sizes were relatively small. The reproducibility of the results across multiple cohorts is more important for the recovery of the ground truth. In order to detect sensitivity, two relatively liberal thresholds (*p* < 0.05, cluster size ≥ 10 voxels; *p* < 0.01, cluster size ≥ 10 voxels) were used. We did not use stringent multiple comparison correction.

### Comparison Between Wavelet–Amplitude of Low-Frequency Fluctuation and Fast Fourier Transform–Amplitude of Low-Frequency Fluctuation

#### Comparison of Sensitivity

To compare the sensitivity between Wavelet-ALFF and FFT-ALFF, we first counted the total number of voxels above the threshold (*p* < 0.05, cluster size ≥ 10 voxels). We then calculated a ratio in each frequency band as follows:

(3) $$r\,a\,t\,i\,o=\frac{n\,u\,m\,b\,e\,r_{\mathrm{Wavelet}\,\text{-}\,\mathrm{ALFF}}}{n\,u\,m\,b\,e\,r_{\mathrm{FFT}\,\text{-}\,\mathrm{ALFF}}}$$

where *number*_Wavelet–ALFF_ denotes the number of voxels detected by Wavelet-ALFF method. Similarly, *number*_*FFT–ALFF*_ denotes the number of voxels detected by FFT-ALFF method. A ratio > 1 implies higher sensitivity for Wavelet-ALFF than FFT-ALFF, and the ratio < 1 implies the opposite.

#### Comparison of Reproducibility Across Cohorts in the ADHD-200 Dataset

As indicated in Section “Subjects and Data Acquisition,” four cohorts from the ADHD-200 open database were available for the current study (Wang et al., 2017). The number of overlapped voxels above the threshold (*p* < 0.05, cluster size ≥ 10 voxels) was applied to represent reproducibility. This implies that, although some voxels showed a significant difference between patients and healthy controls, it is unknown to what extent these brain areas are reproducible in other cohorts. To compare the reproducibility of results between Wavelet-ALFF and FFT-ALFF, we calculated a ratio of each frequency band as follows:

(4) $$r\,a\,t\,i\,o=\frac{o\,v\,e\,r\,l\,a\,p\,p\,e\,d\,n\,u\,m\,b\,e\,r_{W\,a\,v\,e\,l\,e\,t\,\text{-}\,A\,L\,F\,F}}{o\,v\,e\,r\,l\,a\,p\,p\,e\,d\,n\,u\,m\,b\,e\,r_{F\,F\,T\,\text{-}\,A\,L\,F\,F}}$$

where *overlappednumber*_*Wavelet–ALFF*_ denotes the number of overlapped voxels above the threshold (*p* < 0.05, cluster size ≥ 10 voxels) in at least three cohorts in the four ADHD cohorts (NYU, PKU1, PKU2, and PKU3) detected by the Wavelet-ALFF. Similarly, *overlappednumber*_*FFT–ALFF*_ is the number of overlapped voxels of at least three cohorts in the four ADHD cohorts (NYU, PKU1, PKU2, and PKU3) detected by FFT-ALFF. A ratio > 1 implies higher reproducibility for Wavelet-ALFF than FFT-ALFF, and the ratio < 1 implies the opposite.

#### Similarity of Spatial Pattern of Wavelet–Amplitude of Low-Frequency Fluctuation With Fast Fourier Transform–Amplitude of Low-Frequency Fluctuation

We computed the Dice similarity coefficient (DSC) (Dice, 1945; Burunat et al., 2016) to calculate the spatial overlap of Wavelet-ALFF with FFT-ALFF results of each cohort in each frequency band:

(5) $$D\,S\,C=\frac{2\,\left|X\,\cap Y\right|}{\left|X\right|+\left|Y\right|}$$

where *X*, *Y*, and *X*∩*Y* denote the Wavelet-ALFF-based binarized map, corresponding FFT-ALFF-based binarized map, and overlapped map, respectively. Additionally, |⋅| represents the number of voxels in each corresponding map.

## Results

### Comparison of Sensitivity

Table 1 (liberal threshold of *p* < 0.05, cluster size ≥ 10 voxels) lists a comparison of sensitivity between Wavelet-ALFF and FFT-ALFF. A ratio > 1 indicates higher sensitivity for Wavelet-ALFF than FFT-ALFF. It indicated that only seven ratios were < 1 among all 150 ratios (not including mean ratio). It implies that the Wavelet-ALFF indicated higher sensitivity in almost all frequency bands in all cohorts than FFT-ALFF.

**TABLE 1:** Comparison of sensitivity between Wavelet-ALFF and FFT-ALFF [i.e., the ratio, see formula (3) for method] in five cohorts (NYU, PKU1, PKU2, PKU3, and EOEC) by five mother wavelets (db2, bior4.4, morl, meyr, and sym3) in each frequency band.

	db2-ALFF	bior4.4-ALFF	morl-ALFF	meyr-ALFF	sym3-ALFF
**Slow-6 (0–0.0117 Hz)**					
NYU	1.46	1.33	1.25	1.32	1.28
PKU1	1.22	1.15	1.17	1.13	1.16
PKU2	1.37	1.28	1.29	1.36	1.24
PKU3	1.31	1.26	1.26	1.30	1.27
EOEC	1.20	1.18	1.11	1.14	1.17
Mean	1.31	1.24	1.22	1.25	1.22
**Slow-5 (0.0117–0.0273 Hz)**					
NYU	1.12	1.10	1.02	1.07	1.09
PKU1	1.22	1.13	1.05	1.09	1.11
PKU2	1.16	1.08	0.99	1.07	1.09
PKU3	1.25	1.20	1.11	1.19	1.12
EOEC	1.15	1.10	1.01	1.03	1.09
Mean	1.18	1.12	1.04	1.09	1.10
**Slow-4 (0.0273–0.0742 Hz)**					
NYU	1.05	1.03	0.99	1.03	1.03
PKU1	1.11	1.07	1.03	1.06	1.05
PKU2	1.06	1.07	1.05	1.06	1.08
PKU3	1.06	1.06	1.04	1.05	1.08
EOEC	1.04	1.02	0.98	0.99	1.03
Mean	1.06	1.05	1.02	1.04	1.05
**Slow-3 (0.0742–0.1992 Hz)**					
NYU	1.12	1.11	1.07	1.06	1.15
PKU1	1.06	1.07	1.04	1.04	1.08
PKU2	1.03	1.04	1.02	0.99	1.09
PKU3	1.05	1.04	1.05	1.01	1.08
EOEC	1.05	1.06	1.04	1.01	1.09
Mean	1.06	1.06	1.05	1.02	1.10
**Slow-2 (0.1992–0.25 Hz)**					
NYU	1.65	1.43	1.21	1.27	1.61
PKU1	1.57	1.37	1.18	1.20	1.55
PKU2	1.55	1.34	1.20	1.21	1.54
PKU3	1.47	1.34	1.14	1.19	1.49
EOEC	1.47	1.26	1.11	1.12	1.44
Mean	1.54	1.35	1.17	1.20	1.53
**Conventional (0.0117–0.0781 Hz)**					
NYU	1.00	1.00	1.00	1.01	1.00
PKU1	1.08	1.06	1.03	1.06	1.03
PKU2	1.03	1.03	1.02	1.02	1.03
PKU3	1.03	1.03	1.04	1.03	1.02
EOEC	1.01	1.01	0.98	0.98	1.01
Mean	1.03	1.03	1.01	1.02	1.02

*A ratio > 1 indicates more sensitive for Wavelet-ALFF and vice versa. The mean sensitivity across all five cohorts of a given frequency band and a given mother wavelet was also calculated. ALFF, amplitude of low-frequency fluctuation; bior4.4, biorthogonal 4.4; db2, Daubechies 2; FFT, fast Fourier transform; meyr, Meyer; morl, Morlet; sym3, Symlets 3.*

**FIGURE 1:**
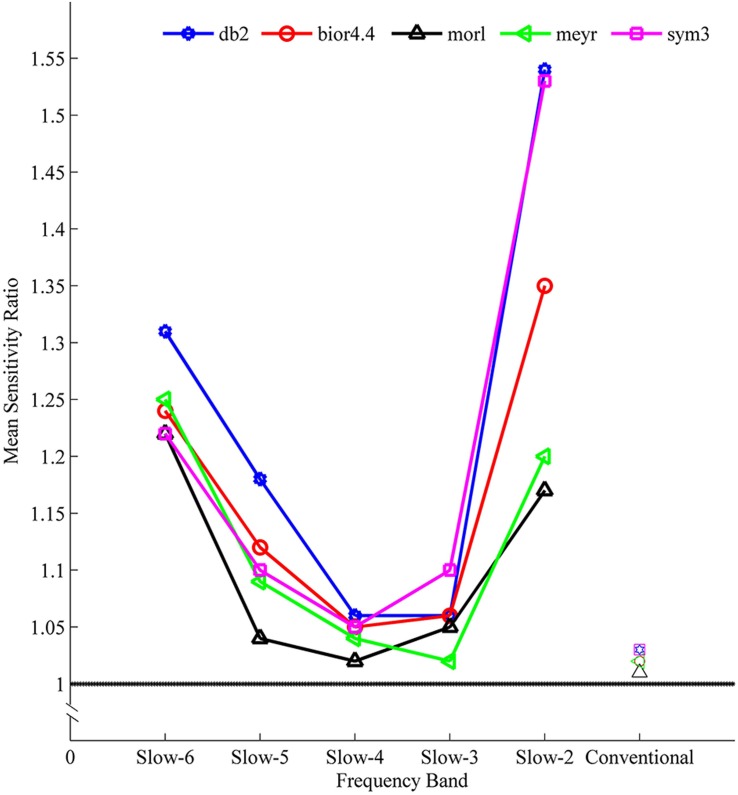
The mean sensitivity (ratio of Wavelet-ALFF to FFT-ALFF) across all five cohorts (NYU, PKU1, PKU2, PKU3, and EOEC) of a given frequency band and a given mother wavelet. All mean ratios were greater than 1, i.e., Wavelet-ALFF was more sensitive than FFT-ALFF. ALFF, amplitude of low-frequency fluctuation; bior4.4, biorthogonal 4.4; db2, Daubechies 2; FFT, fast Fourier transform; meyr, Meyer; morl, Morlet; sym3, Symlets 3.

**FIGURE 2:**
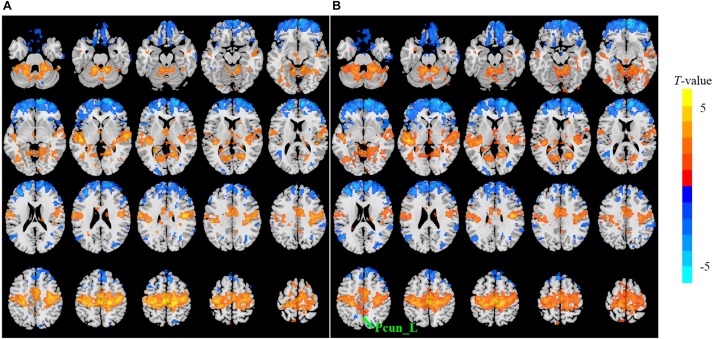
The *t* map (*p* < 0.05, cluster size ≥ 10 voxels) of eyes closed (EC) versus eyes open (EO) in Slow-6 (0–0.0117 Hz) by FFT-ALFF **(A)** and db2-ALFF **(B)**, respectively. Warm colors indicate higher ALFF in EC conditions. ALFF, amplitude of low-frequency fluctuation; FFT, fast Fourier transform; Pcun_L, left precuneus.

**FIGURE 3:**
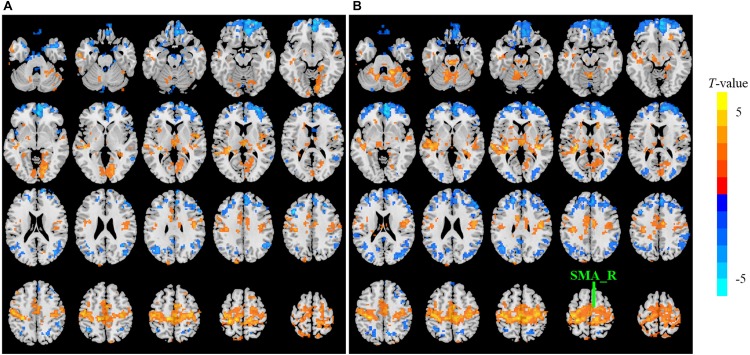
The *t* map (*p* < 0.05, cluster size ≥ 10 voxels) of eyes closed (EC) versus eyes open (EO) in Slow-2 (0.1992–0.25 Hz) by FFT-ALFF **(A)** and db2-ALFF **(B)**, respectively. Warm colors indicate higher ALFF in EC conditions. ALFF, amplitude of low-frequency fluctuation; FFT, fast Fourier transform; SMA_R, right supplementary motor area.

**FIGURE 4:**
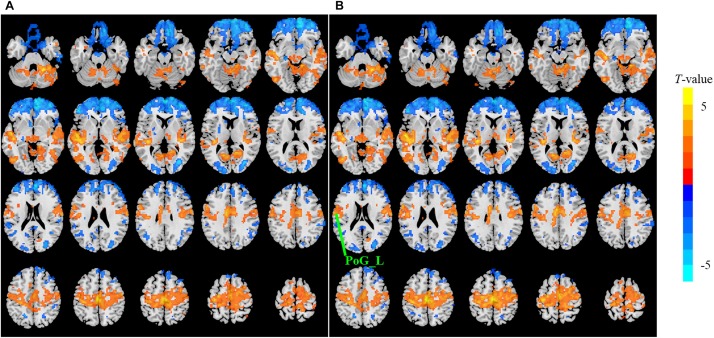
The *t* map (*p* < 0.05, cluster size ≥ 10 voxels) of eyes closed (EC) versus eyes open (EO) in the conventional band (0.0117–0.0781 Hz) by FFT-ALFF **(A)** and db2-ALFF **(B)**, respectively. Warm colors indicate higher ALFF in EC conditions. ALFF, amplitude of low-frequency fluctuation; FFT, fast Fourier transform; PoG_L, left postcentral gyrus.

The sensitivity indicated a frequency-dependent difference for Wavelet-ALFF and FFT-ALFF (Figure 1). Specifically, the sensitivity ratio was the lowest in the conventional frequency band (0.0117–0.0781 Hz). The Wavelet-ALFF in lower frequency band Slow-6 (0–0.0117 Hz) and higher frequency band Slow-2 (0.1992–0.25 Hz) exhibited higher sensitivity than in Slow-3, Slow-4, and Slow-5. In Slow-6, db2 exhibited the highest sensitivity among the five mother wavelets. In Slow-2, db2 and sym3 exhibited higher sensitivity than bior4.4, morl, and meyr. The detailed number of voxels is listed in Supplementary Table S1. The brain areas exhibiting a significant difference between EC and EO in Slow-6, Slow-2, and conventional band are shown in Figures 2–4, respectively. The brain areas exhibiting a significant difference between ADHD and TDC of NYU cohort in Slow-6 and Slow-2 are shown in Supplementary Figures S6,S7, respectively. It should be noted that although there were several maps, we only indicated those of FFT-ALFF and db2-ALFF.

The aforementioned results of higher sensitivity were consistent when a more stringent threshold (*p* < 0.01, cluster size ≥ 10 voxels) was used (Supplementary Tables S2, S3). The mean sensitivity of db2-ALFF results was 2.36 times that of FFT-ALFF in the higher frequency band Slow-2.

### Comparison of Reproducibility Across Cohorts in the ADHD-200 Dataset

**FIGURE 5:**
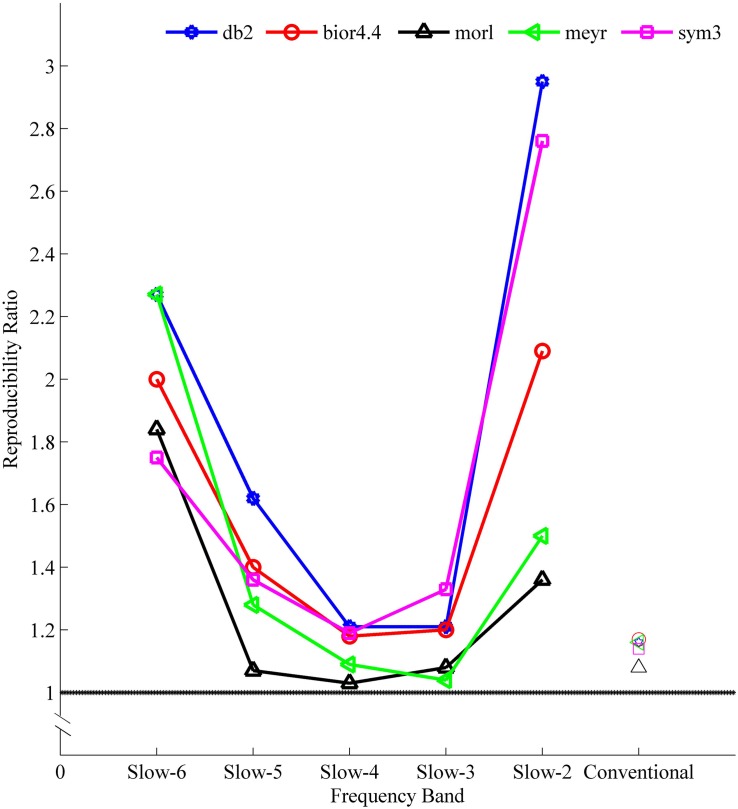
The reproducibility ratio [see formula (4) for method] of Wavelet-ALFF result to FFT-ALFF result. The reproducibility was defined by overlapped voxels of at least three cohorts in the four ADHD cohorts (NYU, PKU1, PKU2, and PKU3). All reproducibility ratios were greater than 1, i.e., Wavelet-ALFF result was more reproducible than FFT-ALFF result. ALFF, amplitude of low-frequency fluctuation; bior4.4, biorthogonal 4.4; db2, Daubechies 2; FFT, fast Fourier transform; meyr, Meyer; morl, Morlet; sym3, Symlets 3.

Figure 5 shows a comparison of reproducibility between Wavelet-ALFF result and FFT-ALFF result. A ratio > 1 indicates higher reproducibility of Wavelet-ALFF than FFT-ALFF. Wavelet-ALFF indicated more reproducible results across cohorts in the ADHD-200 dataset than FFT-ALFF in all frequency bands and all mother wavelets.

The reproducibility indicated a frequency-dependent difference between Wavelet-ALFF results and FFT-ALFF results (Figure 5). Specifically, the reproducibility ratio was the lowest in the conventional frequency band (0.0117–0.0781 Hz). The Wavelet-ALFF in lower frequency band Slow-6 (0–0.0117 Hz) and higher frequency band Slow-2 (0.1992–0.25 Hz) appeared as more reproducible than in Slow-3, Slow-4, and Slow-5. In Slow-6, db2 appeared as the most reproducible, and in Slow-2, db2 and sym3 were more reproducible than bior4.4, morl, and meyr. The detailed number of overlapped voxels of at least three cohorts in the four ADHD cohorts is listed in Supplementary Table S4. The detailed reproducibility ratio is listed in Supplementary Table S5, and the reproducibility brain maps of Slow-6 and Slow-2 are shown in Supplementary Figures S8, S9.

The aforementioned results of higher reproducibility were overall consistent when a more stringent threshold (*p* < 0.01, cluster size ≥ 10 voxels) was used (Supplementary Tables S6, S7). The reproducibility of db2-ALFF results was three times that of FFT-ALFF in the higher frequency band Slow-2.

### Similarity of Spatial Pattern of Wavelet–Amplitude of Low-Frequency Fluctuation With Fast Fourier Transform–Amplitude of Low-Frequency Fluctuation

**FIGURE 6:**
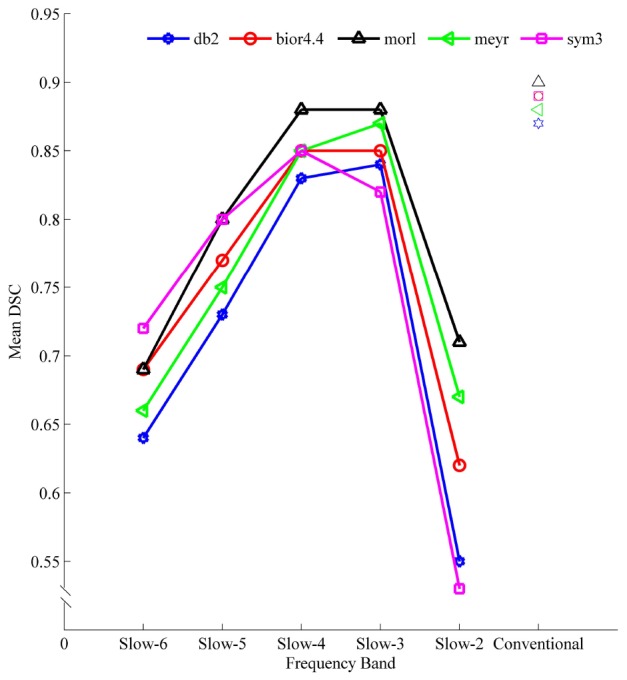
The mean DSC for similarity analysis of spatial pattern calculated from different mother wavelets and FFT-ALFF in different frequency bands. ALFF, amplitude of low-frequency fluctuation; bior4.4, biorthogonal 4.4; db2, Daubechies 2; DSC, Dice similarity coefficient; FFT, fast Fourier transform; meyr, Meyer; morl, Morlet; sym3, Symlets 3.

Dice similarity coefficient indicated a moderate to high similarity (0.53–0.90) of the spatial patterns detected by FFT-ALFF with those detected by Wavelet-ALFF (Figure 6 and Supplementary Table S8). The overlap of Wavelet-ALFF with FFT-ALFF indicated a frequency-dependent character. Specifically, the overlap was less prominent in the higher frequency band Slow-2 (0.1992–0.25 Hz) and Slow-6 (0–0.0117 Hz), while the Slow-3 and Slow-4 indicated a more prominent overlap.

The five mother wavelets were compared, and db2-ALFF and sym3-ALFF exhibited the least prominent overlap with FFT-ALFF in the higher frequency band Slow-2 (0.1992–0.25 Hz).

## Discussion

### Why We Used the EOEC Dataset and the ADHD-200 Dataset

RS-fMRI studies include within-condition, between-condition, and between-group comparisons. Studies on a single condition yielded very robust results of networks. For example, the default mode network can be determined in literally each subject. Among the studies of between-condition comparison within a single group of subjects, differences between EO and EC resting conditions are significantly consistent across studies using FFT-ALFF (Yan et al., 2009; Liu et al., 2013; Zou et al., 2015; Zhao et al., 2018). Most clinical studies correspond to between-group comparison. Although a gold standard does not exist in clinical RS-fMRI studies, reproducibility across different studies is a very important index. Unfortunately, only a very limited number of RS-fMRI studies (e.g., Turner et al., 2013; Wang et al., 2017) tested reproducibility across different datasets. Specifically, ADHD-200 is an open-access dataset and is widely utilized. We recently reported the extremely low reproducibility of RS-fMRI results across ADHD datasets. Therefore, the aim of the current study involved exploring whether Wavelet-ALFF can increase the reproducibility of differences between ADHD and healthy controls across datasets from different research centers.

### Why We Used the Amplitude of Low-Frequency Fluctuation Among the Resting-State Functional MRI Metrics

There are several metrics for RS-fMRI. However, we only used the simplest metric, i.e., ALFF, in the current study. Other metrics involve significantly more options for parameters. For example, there are countless options for seed selection in seed-based functional connectivity analysis. Additionally, most RS-fMRI network metrics are not suitable for coordinate- or voxel-based meta-analysis. ALFF is a typical metric of single-voxel level or single time series analysis. All the previous ALFF studies were based on FFT. Only two studies mentioned WT and used the power as a metric of local activity (Salomon et al., 2011; Bajaj et al., 2013). It should be noted that the ALFF corresponds to the averaged square root of power. Existing studies have not compared the results between FFT-ALFF and Wavelet-ALFF. In the current study, we compared the results of Wavelet-ALFF of five mother wavelets with that of FFT-ALFF on several RS-fMRI cohorts in the conventional frequency band as well as sub-bands. We compared their sensitivity, reproducibility, and overlap.

### Sensitivity and Reproducibility

The sensitivity analysis indicated that Wavelet-ALFF was generally more sensitive than FFT-ALFF to the between-group differences (i.e., ADHD group versus TDC group) and between-condition differences (i.e., EO versus EC) in all frequency bands (Table 1 and Supplementary Table S1). Specifically, db2-ALFF exhibited the highest sensitivity among all the mother wavelets in the very low frequency band Slow-6 (0–0.0117 Hz) and higher frequency band Slow-2 (0.1992–0.25 Hz).

With respect to the reproducibility, as shown in Figure 5 and Supplementary Tables S4, S5, the Wavelet-ALFF results of every mother wavelet were more reproducible across the four ADHD cohorts than the FFT-ALFF results in all frequency bands, while more prominent in Slow-6 (0–0.0117 Hz) and Slow-2 (0.1992–0.25 Hz). Among the five mother wavelets, db2-ALFF exhibited the highest reproducibility. Specifically, for the higher frequency band (Slow-2), db2-ALFF results exhibited a better reproducibility of 2.95 times of FFT-ALFF results (Figure 5 and Supplementary Tables S4, S5).

When a more stringent threshold (*p* < 0.01, cluster size ≥ 10 voxels) was used, the aforementioned results of higher sensitivity and higher reproducibility of Wavelet-ALFF than FFT-ALFF were similarly maintained (Supplementary Tables S2, S3, S6, S7). It should be first noted that high sensitivity does not mean true positive. It merely implies that more voxels were detected. Jia et al. (2018) found that stringent or liberal multiple comparison correction could not control the false discoveries across multiple studies when the effect sizes were relatively small. The reproducibility of the results across multiple cohorts is more important for the recovery of the ground truth. Hence, the reproducibility of the results across four ADHD cohorts was measured. However, higher reproducibility does not always mean a true positive due to the limited number of cohorts. Additional datasets of other disorders should be used in future studies. The current results simply imply that Wavelet-ALFF was slightly better when compared with FFT-ALFF. As shown in the between-condition comparison (i.e., EO versus EC), when compared with those detected by FFT-ALFF, the significantly different voxels detected by db2-ALFF covered more extended physiologically relevant regions albeit similar, such as the precuneus in Slow-6 (Figure 2), supplementary motor area in Slow-2 (Figure 3), and postcentral gyrus in the conventional band (Figure 4), which can be related to alpha waves modulated by the closure of eyes. In the between-group comparison (i.e., ADHD group versus TDC group) for NYU cohort, when compared with FFT-ALFF, db2-ALFF detected more regions that can be associated with ADHD pathophysiology such as the middle frontal gyrus and middle occipital gyrus in Slow-6 (Supplementary Figure S6), superior occipital gyrus, and supplementary motor area in Slow-2 (Supplementary Figure S7). Nevertheless, future studies should focus on the same.

There are two reasons for the superiority of db2 compared to the other mother wavelets. First, the support width of db2 is less than that of the other mother wavelets, and the less support width of db2 makes an increase in the degree of localization of the wavelet coefficients (Zhang et al., 2016), which makes db2 more effective in detecting local or non-stationary features of the signal. Second, db2 appears to be quite similar to the hemodynamic response function (HRF) among the five mother wavelets. A CWT of HRF was implemented with a frequent parameter of 64 (2^4^) scale. If the summation of the absolute value of the wavelet coefficients is larger, the similarity is higher (Rafiee et al., 2011). It was found that the summation of db2 (229.4348) was the largest compared to sym3 (224.7303), bior4.4 (219.8648), meyr (201.7399), and morl (182.8433). Higher similarities may facilitate identification of the signal feature more precisely (Singh and Tiwari, 2006; Rafiee et al., 2011; Ngui et al., 2013).

### Very Low Frequency Band Slow-6 and Higher Frequency Band Slow-2

In RS-fMRI studies, the very low frequency band Slow-6 has attracted less attention. Zhang et al. (2015) indicated that the FFT-ALFF of Slow-6 (0–0.0117 Hz) in the basal ganglia was higher during the state of real feedback finger force than during sham feedback state. Beyond the conventional low frequency band (<0.1 Hz), several RS-fMRI studies investigated the higher frequency band (>0.1 Hz) signal. Yuan et al. (2014) used fast sampling (TR = 400 ms) RS-fMRI and suggested that the differences of fluctuation amplitude between EO and EC resting states were in the conventional frequency band (<0.1 Hz) and higher frequency band (up to 0.35 Hz). Two independent RS-fMRI studies on chronic pain used conventional sampling rate (TR = 2 s) and consistently found increased spectral power of patients in the higher frequency band (Malinen et al., 2010; Otti et al., 2013). It should be noted that the amplitude is proportional to the square root of spectral power (Zang et al., 2007). The aforementioned studies all suggested that the very low frequency band Slow-6 and higher frequency band Slow-2 can contain neural-related information. We recommend db2-ALFF to substitute FFT-ALFF in future studies given its higher performance in sensitivity and reproducibility in very low frequency ALFF and higher frequency ALFF of db2 mother wavelet than FFT-ALFF. However, there is no widely accepted gold standard for RS-fMRI results of between- or within-group comparison studies. The current better reproducibility of db2-ALFF when compared to other mother wavelets and FFT-ALFF was obtained in only four ADHD cohorts. Therefore, this should be tested in more datasets in the future.

It should be noted that although db2-ALFF exhibited higher sensitivity and higher reproducibility in the very low frequency and higher frequency band, it does not imply that the very low frequency band and higher frequency band are more physiologically or pathophysiologically meaningful than the conventional frequency band. Specifically, the conventional frequency band detected the largest number of voxels among sub-frequency bands (Supplementary Figure S10). The results simply imply that the performance of db2-ALFF exceeds that of FFT-ALFF.

### Overlap of Spatial Pattern by Wavelet–Amplitude of Low-Frequency Fluctuation With Fast Fourier Transform–Amplitude of Low-Frequency Fluctuation

The vast majority of previous ALFF studies have used FFT, and thus we calculated the overlap of spatial pattern of Wavelet-ALFF results with FFT-ALFF results. Generally, the spatial patterns detected by FFT-ALFF with Wavelet-ALFF were very similar in the conventional frequency band (0.0117–0.0781 Hz), Slow-3 (0.0742–0.1992 Hz), and Slow-4 (0.0273–0.0742 Hz) with a DSC of approximately 0.85. The overlap was less prominent in the higher frequency band Slow-2 (0.1992–0.25 Hz) and very low frequency band Slow-6 (0–0.0117 Hz), wherein the db2-ALFF and sym3-ALFF in the higher frequency band Slow-2 (0.1992–0.25 Hz) exhibited less spatial overlap with FFT-ALFF than all other conditions (Figure 6). The lower spatial overlap of results by db2- and sym3-ALFF with those of FFT-ALFF could be attributed to the improved performance of both sensitivity and reproducibility of db2- and sym3-ALFF.

### Limitations

First, the study used only five mother wavelets that were used in previous fMRI literature. Future studies should use more mother wavelets. Second, the dynamic character is an advantage of WT. However, there were an excessive number of time points, and thus it is difficult to interpret the results if the *t*-test was performed on every time point. A future study should propose an integrated metric to characterize its dynamics. Third, db2-ALFF indicated optimal sensitivity and reproducibility in higher frequency Slow-2. However, based on the Shannon–Nyquist sampling theorem, the lower sampling rate (TR = 2 s in the current study) resulted in aliasing effect. This implies that the signal in Slow-2 can be aliased from higher frequency physiological noise of heart beating (around 1.2 Hz) and respiratory (around 0.33 Hz). Future studies could use db2-ALFF in fast sampling rate RS-fMRI dataset. Fourth, we used only four independent datasets of ADHD cohorts to test the reproducibility. It should be noted that higher reproducibility of Wavelet-ALFF in such small number of cohorts does not imply higher specificity. The current results should be tested in more (preferably more than 20) datasets. Fifth, the current study compared only an RS-fMRI metric, i.e., ALFF. Systematic investigations should be performed for other metrics to compare wavelet- and FFT-based analyses. Sixth, many preprocessing parameters may affect the results. Additional datasets of other brain disorders are also important. Future studies will focus on these aspects.

## Conclusion

In summary, the results indicated that Wavelet-ALFF was generally more sensitive to the between-group and between-condition differences than FFT-ALFF in all frequency bands. More importantly, the Wavelet-ALFF results indicated a better reproducibility across the four ADHD cohorts than the FFT-ALFF results in all frequency bands. Specifically, in the higher frequency band Slow-2 (0.1992–0.25 Hz), the reproducibility of db2-ALFF result was 2.95 times that of FFT-ALFF result. This suggested that Wavelet-ALFF can replace FFT-ALFF as a potentially reliable marker to determine the exact location of local abnormal brain activity in future studies and further help precise intervention such as deep brain stimulation and transcranial magnetic stimulation.

## Data Availability Statement

The datasets generated for this study are available on request to the corresponding author.

## Ethics Statement

The studies involving human participants were reviewed and approved by the Ethics Committee of the Center for Cognition and Brain Disorders (CCBD) at Hangzhou Normal University; the Research Ethics Review Board of Peking University Institute of Mental Health; the University Committee on Activities Involving Human Subjects at New York University. Written informed consent to participate in this study was provided by the participants’ legal guardian/next of kin.

## Author Contributions

Y-FZ and MZ proposed and supervised the project. F-FL and Y-FZ designed the algorithm, analyzed the data, and wrote the manuscript. J-BW, L-XY, and Z-WZ collected the data and reviewed the process. HX and S-HM revised and commented on the manuscript.

## Conflict of Interest

The authors declare that the research was conducted in the absence of any commercial or financial relationships that could be construed as a potential conflict of interest.

## Footnotes

1 http://fcon_1000.projects.nitrc.org/indi/adhd200/

2 http://www.fil.ion.ucl.ac.uk/spm/software/spm8/
