# Supplementary material for: Higher Sensitivity and Reproducibility of Wavelet-Based Amplitude of Resting-State fMRI
Source: Front Neurosci. 2020 Mar 31;14:224. doi: 10.3389/fnins.2020.00224 (PMC7145399; doi:10.3389/fnins.2020.00224)
Supplement: Supplementary file 1 [file Data_Sheet_1.doc]

***Supplementary Material***

**Supplementary Table S1**

The number of voxels showing significant difference (*p* < 0.05, cluster size ≥10 voxels) in EOEC dataset (EOEC) and four cohorts of ADHD (NYU, PKU1, PKU2, and PKU3) calculated from Wavelet-ALFF and FFT-ALFF results in different frequency bands.

|  |  | db2-ALFF | bior4.4-ALFF | morl-ALFF | meyr-ALFF | sym3-ALFF | FFT-ALFF |
| --- | --- | --- | --- | --- | --- | --- | --- |
| Slow-6  (0 - 0.0117 Hz) | NYU | 5299 | 4821 | 4521 | 4796 | 4629 | 3621 |
|  | PKU1 | 4284 | 4020 | 4092 | 3946 | 4056 | 3501 |
|  | PKU2 | 5926 | 5561 | 5579 | 5889 | 5356 | 4336 |
|  | PKU3 | 3287 | 3164 | 3180 | 3266 | 3194 | 2517 |
|  | EOEC | 15362 | 15071 | 14220 | 14537 | 15016 | 12789 |
| Slow-5  (0.0117 - 0.0273 Hz) | NYU | 6645 | 6506 | 6045 | 6335 | 6457 | 5925 |
|  | PKU1 | 5333 | 4938 | 4606 | 4792 | 4869 | 4388 |
|  | PKU2 | 8240 | 7665 | 7037 | 7561 | 7679 | 7074 |
|  | PKU3 | 4087 | 3905 | 3633 | 3893 | 3657 | 3266 |
|  | EOEC | 15081 | 14361 | 13287 | 13492 | 14303 | 13103 |
| Slow-4  (0.0273 - 0.0742 Hz) | NYU | 7245 | 7062 | 6798 | 7064 | 7094 | 6885 |
|  | PKU1 | 7124 | 6866 | 6614 | 6845 | 6760 | 6443 |
|  | PKU2 | 8983 | 9068 | 8917 | 8991 | 9170 | 8500 |
|  | PKU3 | 4193 | 4192 | 4101 | 4172 | 4255 | 3957 |
|  | EOEC | 15964 | 15678 | 14984 | 15184 | 15815 | 15313 |
| Slow-3  (0.0742 - 0.1992 Hz) | NYU | 6672 | 6638 | 6405 | 6317 | 6893 | 5968 |
|  | PKU1 | 7965 | 8002 | 7799 | 7798 | 8061 | 7482 |
|  | PKU2 | 7032 | 7057 | 6950 | 6773 | 7458 | 6811 |
|  | PKU3 | 3719 | 3691 | 3727 | 3588 | 3844 | 3552 |
|  | EOEC | 14214 | 14357 | 14173 | 13722 | 14780 | 13582 |
| Slow-2  (0.1992 - 0.25 Hz) | NYU | 5608 | 4873 | 4110 | 4311 | 5474 | 3406 |
|  | PKU1 | 6643 | 5801 | 4993 | 5063 | 6579 | 4233 |
|  | PKU2 | 5831 | 5049 | 4492 | 4555 | 5788 | 3754 |
|  | PKU3 | 3240 | 2946 | 2516 | 2620 | 3273 | 2202 |
|  | EOEC | 11625 | 9940 | 8755 | 8866 | 11374 | 7896 |
| Conventional  (0.0117 - 0.0781 Hz) | NYU | 7294 | 7254 | 7271 | 7312 | 7252 | 7274 |
|  | PKU1 | 6799 | 6658 | 6457 | 6634 | 6459 | 6268 |
|  | PKU2 | 9118 | 9151 | 9058 | 9060 | 9165 | 8880 |
|  | PKU3 | 4384 | 4378 | 4410 | 4389 | 4322 | 4247 |
|  | EOEC | 16396 | 16302 | 15943 | 15820 | 16435 | 16195 |

**Supplementary Table S2**

The number of voxels showing significant difference with a more stringent threshold (*p* < 0.01, cluster size ≥10 voxels) in EOEC dataset (EOEC) and four cohorts of ADHD (NYU, PKU1, PKU2, and PKU3) calculated from Wavelet-ALFF and FFT-ALFF results in the different frequency bands.

|  |  | db2-ALFF | bior4.4-ALFF | morl-ALFF | meyr-ALFF | sym3-ALFF | FFT-ALFF |
| --- | --- | --- | --- | --- | --- | --- | --- |
| Slow-6  (0 - 0.0117 Hz) | NYU | 1349 | 1231 | 1065 | 1238 | 1170 | 740 |
|  | PKU1 | 878 | 749 | 703 | 702 | 773 | 465 |
|  | PKU2 | 1347 | 1194 | 1368 | 1281 | 1134 | 843 |
|  | PKU3 | 649 | 581 | 563 | 624 | 516 | 334 |
|  | EOEC | 6961 | 6876 | 5924 | 6529 | 6693 | 5030 |
| Slow-5  (0.0117 - 0.0273 Hz) | NYU | 2213 | 2052 | 1800 | 2004 | 1925 | 1696 |
|  | PKU1 | 1292 | 1236 | 1173 | 1150 | 1244 | 1063 |
|  | PKU2 | 2522 | 2229 | 1865 | 2145 | 2190 | 1812 |
|  | PKU3 | 924 | 872 | 704 | 794 | 802 | 607 |
|  | EOEC | 6267 | 5745 | 4969 | 5202 | 5702 | 4870 |
| Slow-4  (0.0273 - 0.0742 Hz) | NYU | 2485 | 2355 | 2253 | 2371 | 2423 | 2247 |
|  | PKU1 | 1986 | 1910 | 1744 | 1818 | 1745 | 1583 |
|  | PKU2 | 3310 | 3362 | 3244 | 3354 | 3309 | 2984 |
|  | PKU3 | 921 | 900 | 838 | 866 | 970 | 828 |
|  | EOEC | 7445 | 7233 | 6725 | 6941 | 7252 | 6968 |
| Slow-3  (0.0742 - 0.1992 Hz) | NYU | 2164 | 2081 | 2022 | 1955 | 2213 | 1821 |
|  | PKU1 | 2147 | 2177 | 2140 | 2107 | 2318 | 2002 |
|  | PKU2 | 2047 | 2021 | 1908 | 1807 | 2277 | 1839 |
|  | PKU3 | 747 | 706 | 717 | 670 | 838 | 647 |
|  | EOEC | 6278 | 6268 | 5995 | 5692 | 6707 | 5628 |
| Slow-2  (0.1992 - 0.25 Hz) | NYU | 1499 | 1162 | 834 | 902 | 1431 | 448 |
|  | PKU1 | 1538 | 1247 | 987 | 1010 | 1528 | 782 |
|  | PKU2 | 1305 | 979 | 850 | 824 | 1182 | 566 |
|  | PKU3 | 637 | 500 | 364 | 373 | 599 | 303 |
|  | EOEC | 4142 | 3019 | 2378 | 2499 | 3972 | 1995 |
| Conventional  (0.0117 - 0.0781 Hz) | NYU | 2588 | 2585 | 2493 | 2557 | 2570 | 2480 |
|  | PKU1 | 1782 | 1724 | 1659 | 1749 | 1670 | 1579 |
|  | PKU2 | 3289 | 3296 | 3197 | 3240 | 3246 | 3149 |
|  | PKU3 | 989 | 1005 | 971 | 990 | 1024 | 952 |
|  | EOEC | 7610 | 7484 | 7199 | 7223 | 7405 | 7305 |

**Supplementary Table S3**

Comparison of sensitivity between Wavelet-ALFF and FFT-ALFF (i.e., the ratio of Wavelet-ALFF to FFT-ALFF, see formula (3) for method) with a more stringent threshold (*p* < 0.01, cluster size ≥10 voxels) in 5 cohorts (NYU, PKU1, PKU2, PKU3, and EOEC) by 5 mother wavelets (db2, bior4.4, morl, meyr, and sym3) in each frequency band. A ratio > 1 indicates higher sensitivity for Wavelet-ALFF and vice versa.

|  |  | db2-ALFF | bior4.4-ALFF | morl-ALFF | meyr-ALFF | sym3-ALFF |
| --- | --- | --- | --- | --- | --- | --- |
| Slow-6  (0 - 0.0117 Hz) | NYU | 1.82 | 1.66 | 1.44 | 1.67 | 1.58 |
|  | PKU1 | 1.89 | 1.61 | 1.51 | 1.51 | 1.66 |
|  | PKU2 | 1.60 | 1.42 | 1.62 | 1.52 | 1.35 |
|  | PKU3 | 1.94 | 1.74 | 1.69 | 1.87 | 1.54 |
|  | EOEC | 1.38 | 1.37 | 1.18 | 1.30 | 1.33 |
|  | Mean | 1.73 | 1.56 | 1.49 | 1.57 | 1.49 |
| Slow-5  (0.0117 - 0.0273 Hz) | NYU | 1.30 | 1.21 | 1.06 | 1.18 | 1.14 |
|  | PKU1 | 1.22 | 1.16 | 1.10 | 1.08 | 1.17 |
|  | PKU2 | 1.39 | 1.23 | 1.03 | 1.18 | 1.21 |
|  | PKU3 | 1.52 | 1.44 | 1.16 | 1.31 | 1.32 |
|  | EOEC | 1.29 | 1.18 | 1.02 | 1.07 | 1.17 |
|  | Mean | 1.34 | 1.24 | 1.07 | 1.16 | 1.20 |
| Slow-4  (0.0273 - 0.0742 Hz) | NYU | 1.11 | 1.05 | 1.00 | 1.06 | 1.08 |
|  | PKU1 | 1.25 | 1.21 | 1.10 | 1.15 | 1.10 |
|  | PKU2 | 1.11 | 1.13 | 1.09 | 1.12 | 1.11 |
|  | PKU3 | 1.11 | 1.09 | 1.01 | 1.05 | 1.17 |
|  | EOEC | 1.07 | 1.04 | 0.97 | 1.00 | 1.04 |
|  | Mean | 1.13 | 1.10 | 1.03 | 1.07 | 1.10 |
| Slow-3  (0.0742 - 0.1992 Hz) | NYU | 1.19 | 1.14 | 1.11 | 1.07 | 1.22 |
|  | PKU1 | 1.07 | 1.09 | 1.07 | 1.05 | 1.16 |
|  | PKU2 | 1.11 | 1.10 | 1.04 | 0.98 | 1.24 |
|  | PKU3 | 1.15 | 1.09 | 1.11 | 1.04 | 1.30 |
|  | EOEC | 1.12 | 1.11 | 1.07 | 1.01 | 1.19 |
|  | Mean | 1.13 | 1.11 | 1.08 | 1.03 | 1.22 |
| Slow-2  (0.1992 - 0.25 Hz) | NYU | 3.35 | 2.59 | 1.86 | 2.01 | 3.19 |
|  | PKU1 | 1.97 | 1.59 | 1.26 | 1.29 | 1.95 |
|  | PKU2 | 2.31 | 1.73 | 1.50 | 1.46 | 2.09 |
|  | PKU3 | 2.10 | 1.65 | 1.20 | 1.23 | 1.98 |
|  | EOEC | 2.08 | 1.51 | 1.19 | 1.25 | 1.99 |
|  | Mean | 2.36 | 1.82 | 1.40 | 1.45 | 2.24 |
| Conventional  (0.0117 - 0.0781 Hz) | NYU | 1.04 | 1.04 | 1.01 | 1.03 | 1.04 |
|  | PKU1 | 1.13 | 1.09 | 1.05 | 1.11 | 1.06 |
|  | PKU2 | 1.04 | 1.05 | 1.02 | 1.03 | 1.03 |
|  | PKU3 | 1.04 | 1.06 | 1.02 | 1.04 | 1.08 |
|  | EOEC | 1.04 | 1.02 | 0.99 | 0.99 | 1.01 |
|  | Mean | 1.06 | 1.05 | 1.02 | 1.04 | 1.04 |

**Supplementary Table S4**

The numbers of overlapped voxels of at least 3 cohorts calculated from Wavelet-ALFF (db2, bior4.4, morl, meyr and sym3) and FFT-ALFF results (*p* < 0.05, cluster size ≥10 voxels).

|  | Slow-6  (0 - 0.0117 Hz) | Slow-5  (0.0117 - 0.0273 Hz) | Slow-4  (0.0273 - 0.0742 Hz) | Slow-3  (0.0742 - 0.1992 Hz) | Slow-2  (0.1992 - 0.25 Hz) | Convention  (0.0117 - 0.0781 Hz) |
| --- | --- | --- | --- | --- | --- | --- |
| db2-ALFF | 184 | 378 | 457 | 379 | 218 | 513 |
| bior4.4-ALFF | 162 | 328 | 444 | 377 | 155 | 514 |
| morl-ALFF | 149 | 250 | 389 | 339 | 101 | 478 |
| meyr-ALFF | 184 | 299 | 412 | 324 | 111 | 510 |
| sym3-ALFF | 142 | 318 | 447 | 415 | 204 | 503 |
| FFT-ALFF | 81 | 234 | 377 | 313 | 74 | 441 |

**Supplementary Table S5**

The reproducibility ratio (see formula (4) for method) of Wavelet-ALFF result to FFT-ALFF result (*p* < 0.05, cluster size ≥10 voxels). The reproducibility was defined by overlapped voxels of at least 3 cohorts in the 4 ADHD cohorts (NYU, PKU1, PKU2, and PKU3). All reproducibility ratios were greater than 1, i.e., Wavelet-ALFF result was more reproducible than FFT-ALFF result.

|  | Slow-6  (0 - 0.0117 Hz) | Slow-5  (0.0117 - 0.0273 Hz) | Slow-4  (0.0273 - 0.0742 Hz) | Slow-3  (0.0742 - 0.1992 Hz) | Slow-2  (0.1992 - 0.25 Hz) | Conventional  (0.0117 - 0.0781 Hz) |
| --- | --- | --- | --- | --- | --- | --- |
| db2-ALFF | 2.27 | 1.62 | 1.21 | 1.21 | 2.95 | 1.16 |
| bior4.4-ALFF | 2.00 | 1.40 | 1.18 | 1.20 | 2.09 | 1.17 |
| morl-ALFF | 1.84 | 1.07 | 1.03 | 1.08 | 1.36 | 1.08 |
| meyr-ALFF | 2.27 | 1.28 | 1.09 | 1.04 | 1.50 | 1.16 |
| sym3-ALFF | 1.75 | 1.36 | 1.19 | 1.33 | 2.76 | 1.14 |

**Supplementary Table S6**

The numbers of overlapped voxels of at least 3 cohorts calculated from Wavelet-ALFF (db2, bior4.4, morl, meyr and sym3) and FFT-ALFF results with a more stringent threshold (*p* < 0.01, cluster size ≥10 voxels).

|  | Slow-6  (0 - 0.0117 Hz) | Slow-5  (0.0117 - 0.0273 Hz) | Slow-4  (0.0273 - 0.0742 Hz) | Slow-3  (0.0742 - 0.1992 Hz) | Slow-2  (0.1992 - 0.25 Hz) | Convention  (0.0117 - 0.0781 Hz) |
| --- | --- | --- | --- | --- | --- | --- |
| db2-ALFF | 11 | 21 | 43 | 23 | 6 | 41 |
| bior4.4-ALFF | 8 | 20 | 32 | 25 | 0 | 40 |
| morl-ALFF | 0 | 14 | 32 | 14 | 0 | 34 |
| meyr-ALFF | 4 | 14 | 32 | 12 | 0 | 39 |
| sym3-ALFF | 6 | 16 | 30 | 37 | 7 | 40 |
| FFT-ALFF | 2 | 6 | 17 | 13 | 2 | 37 |

**Supplementary Table S7**

The reproducibility ratio (see formula (4) for method) of Wavelet-ALFF result to FFT-ALFF result wih a more stringent threshold (*p* < 0.01, cluster size ≥10 voxels). The reproducibility was defined by overlapped voxels of at least 3 cohorts in the 4 ADHD cohorts (NYU, PKU1, PKU2, and PKU3). Most of the reproducibility ratios were greater than 1, i.e., Wavelet-ALFF result was more reproducible than FFT-ALFF result.

|  | Slow-6  (0 - 0.0117 Hz) | Slow-5  (0.0117 - 0.0273 Hz) | Slow-4  (0.0273 - 0.0742 Hz) | Slow-3  (0.0742 - 0.1992 Hz) | Slow-2  (0.1992 - 0.25 Hz) | Conventional  (0.0117 - 0.0781 Hz) |
| --- | --- | --- | --- | --- | --- | --- |
| db2-ALFF | 5.50 | 3.50 | 2.53 | 1.77 | 3.00 | 1.11 |
| bior4.4-ALFF | 4.00 | 3.33 | 1.88 | 1.92 | 0.00 | 1.08 |
| morl-ALFF | 0.00 | 2.33 | 1.88 | 1.08 | 0.00 | 0.92 |
| meyr-ALFF | 2.00 | 2.33 | 1.88 | 0.92 | 0.00 | 1.05 |
| sym3-ALFF | 3.00 | 2.67 | 1.76 | 2.85 | 3.50 | 1.08 |

**Supplementary Table S8**

The Dice similarity coefficient (DSC) between Wavelet-ALFF and FFT-ALFF results (*p* < 0.05, cluster size ≥10 voxels) of each cohort in each frequency band and the mean DSC of different datasets calculated from the same mother wavelet and FFT-ALFF in the same frequency band.

|  |  | db2-ALFF  &  FFT-ALFF | bior4.4-ALFF &  FFT-ALFF | morl-ALFF  &  FFT-ALFF | meyr-ALFF  &  FFT-ALFF | sym3-ALFF  &  FFT-ALFF |
| --- | --- | --- | --- | --- | --- | --- |
| Slow-6 (0 - 0.0117 Hz) | NYU | 0.62 | 0.67 | 0.67 | 0.63 | 0.70 |
|  | PKU1 | 0.60 | 0.64 | 0.64 | 0.60 | 0.69 |
|  | PKU2 | 0.60 | 0.66 | 0.67 | 0.63 | 0.69 |
|  | PKU3 | 0.62 | 0.67 | 0.66 | 0.63 | 0.70 |
|  | EOEC | 0.78 | 0.80 | 0.81 | 0.79 | 0.82 |
|  | Mean | 0.64 | 0.69 | 0.69 | 0.66 | 0.72 |
| Slow-5 (0.0117 - 0.0273 Hz) | NYU | 0.73 | 0.77 | 0.80 | 0.75 | 0.80 |
|  | PKU1 | 0.67 | 0.74 | 0.78 | 0.72 | 0.77 |
|  | PKU2 | 0.74 | 0.79 | 0.81 | 0.76 | 0.82 |
|  | PKU3 | 0.69 | 0.73 | 0.76 | 0.70 | 0.76 |
|  | EOEC | 0.80 | 0.83 | 0.84 | 0.81 | 0.84 |
|  | Mean | 0.73 | 0.77 | 0.80 | 0.75 | 0.80 |
| Slow-4 (0.0273 - 0.0742 Hz) | NYU | 0.83 | 0.85 | 0.87 | 0.84 | 0.84 |
|  | PKU1 | 0.80 | 0.84 | 0.87 | 0.83 | 0.84 |
|  | PKU2 | 0.85 | 0.87 | 0.90 | 0.87 | 0.86 |
|  | PKU3 | 0.79 | 0.82 | 0.85 | 0.81 | 0.81 |
|  | EOEC | 0.88 | 0.89 | 0.90 | 0.89 | 0.88 |
|  | Mean | 0.83 | 0.85 | 0.88 | 0.85 | 0.85 |
| Slow-3 (0.0742 - 0.1992 Hz) | NYU | 0.83 | 0.85 | 0.86 | 0.86 | 0.81 |
|  | PKU1 | 0.85 | 0.86 | 0.88 | 0.87 | 0.83 |
|  | PKU2 | 0.85 | 0.86 | 0.88 | 0.88 | 0.82 |
|  | PKU3 | 0.81 | 0.83 | 0.86 | 0.85 | 0.78 |
|  | EOEC | 0.85 | 0.87 | 0.89 | 0.88 | 0.85 |
|  | Mean | 0.84 | 0.85 | 0.88 | 0.87 | 0.82 |
| Slow-2 (0.1992 - 0.25 Hz) | NYU | 0.51 | 0.57 | 0.66 | 0.61 | 0.49 |
|  | PKU1 | 0.58 | 0.66 | 0.75 | 0.72 | 0.58 |
|  | PKU2 | 0.58 | 0.66 | 0.74 | 0.71 | 0.57 |
|  | PKU3 | 0.47 | 0.54 | 0.64 | 0.60 | 0.46 |
|  | EOEC | 0.59 | 0.67 | 0.76 | 0.73 | 0.57 |
|  | Mean | 0.55 | 0.62 | 0.71 | 0.67 | 0.53 |
| Conventional (0.0117 - 0.0781 Hz) | NYU | 0.88 | 0.89 | 0.90 | 0.89 | 0.89 |
|  | PKU1 | 0.85 | 0.87 | 0.89 | 0.86 | 0.88 |
|  | PKU2 | 0.89 | 0.90 | 0.92 | 0.90 | 0.90 |
|  | PKU3 | 0.84 | 0.86 | 0.88 | 0.85 | 0.85 |
|  | EOEC | 0.91 | 0.91 | 0.92 | 0.91 | 0.91 |
|  | Mean | 0.87 | 0.89 | 0.90 | 0.88 | 0.89 |
