# Supplementary material for: Higher Sensitivity and Reproducibility of Wavelet-Based Amplitude of Resting-State fMRI
Source: Front Neurosci. 2020 Mar 31;14:224. doi: 10.3389/fnins.2020.00224 (PMC7145399; doi:10.3389/fnins.2020.00224)
Supplement: Supplementary file 2 [file Data_Sheet_2.pdf]

Supplementary Material

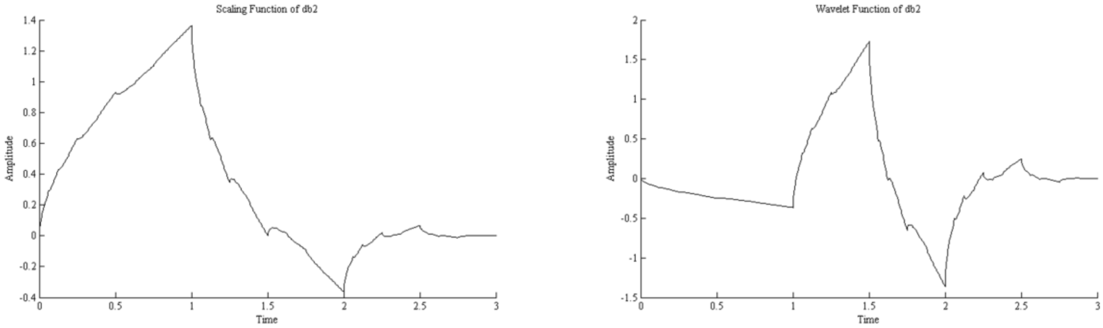

Supplementary Figure S1. The scaling function and wavelet function of Daubechies2 (db2).

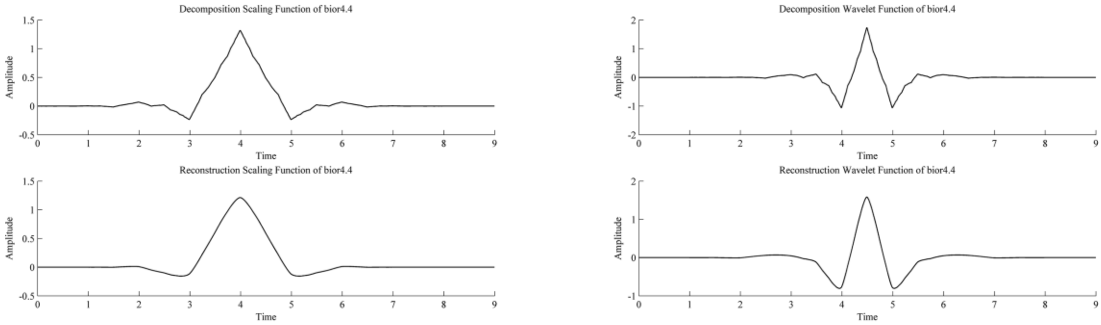

Supplementary Figure S2. The decomposition scaling function, decomposition wavelet function, reconstruction scaling function, and reconstruction wavelet function of biorthogonal 4.4 (bior 4.4).

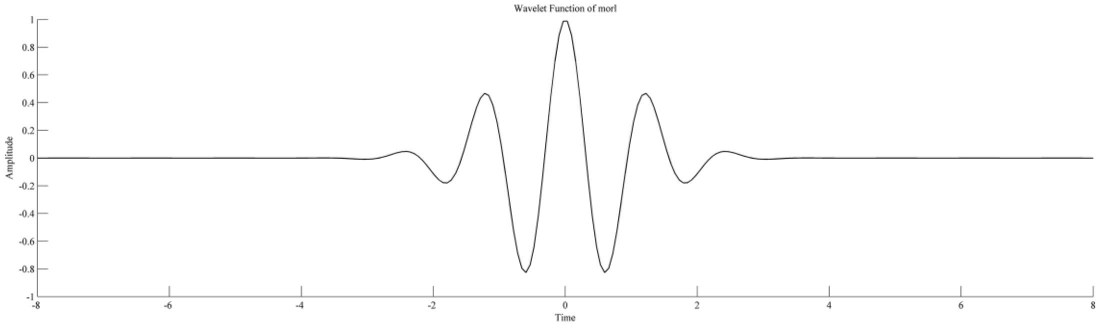

Supplementary Figure S3. The wavelet function of Morlet (morl).

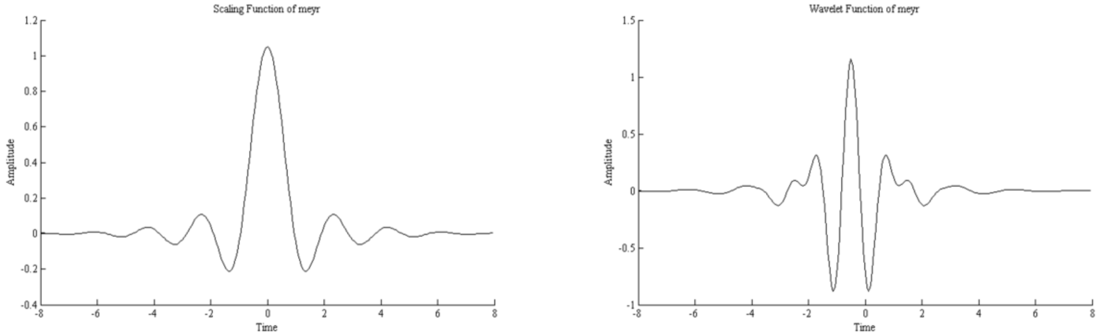

Supplementary Figure S4. The scaling function and wavelet function of Meyer (meyr).

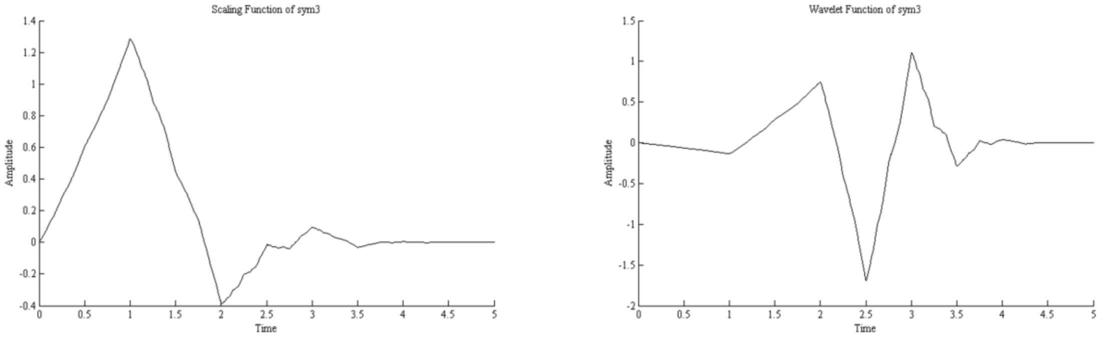

Supplementary Figure S5. The scaling function and wavelet function of Symlets 3 (sym3).

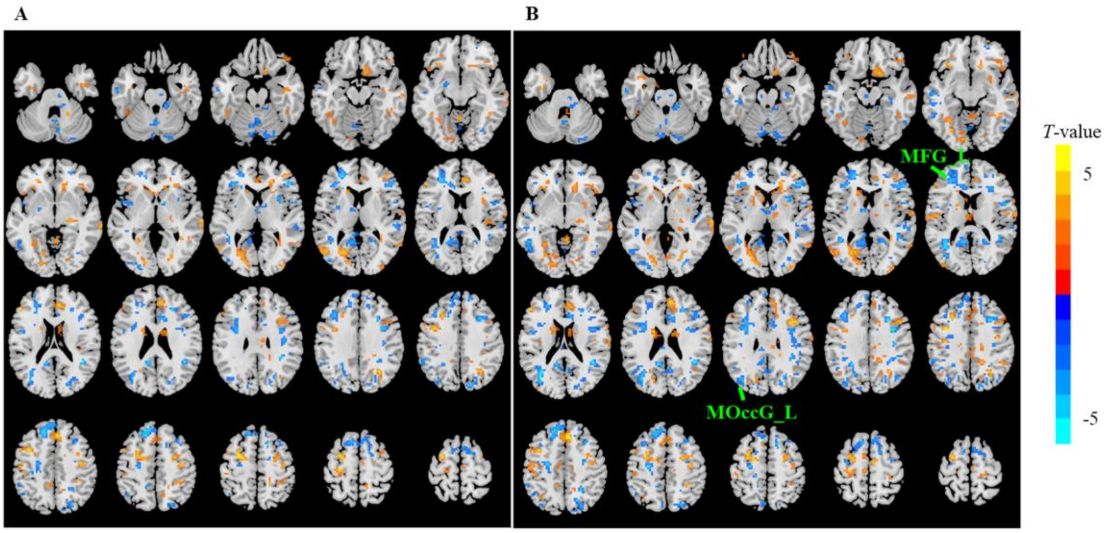

Supplementary Figure S6. The *t* map ($p < 0.05$, cluster size ≥ 10 voxels) of ADHD vs. TDC of NYU cohort in Slow-6 (0 - 0.0117 Hz) by FFT-ALFF **(A)** and db2-ALFF **(B)**, respectively. Cold colors indicate lower ALFF in ADHD. MFG_L, left middle frontal gyrus; MOccG_L, left middle occipital gyrus.

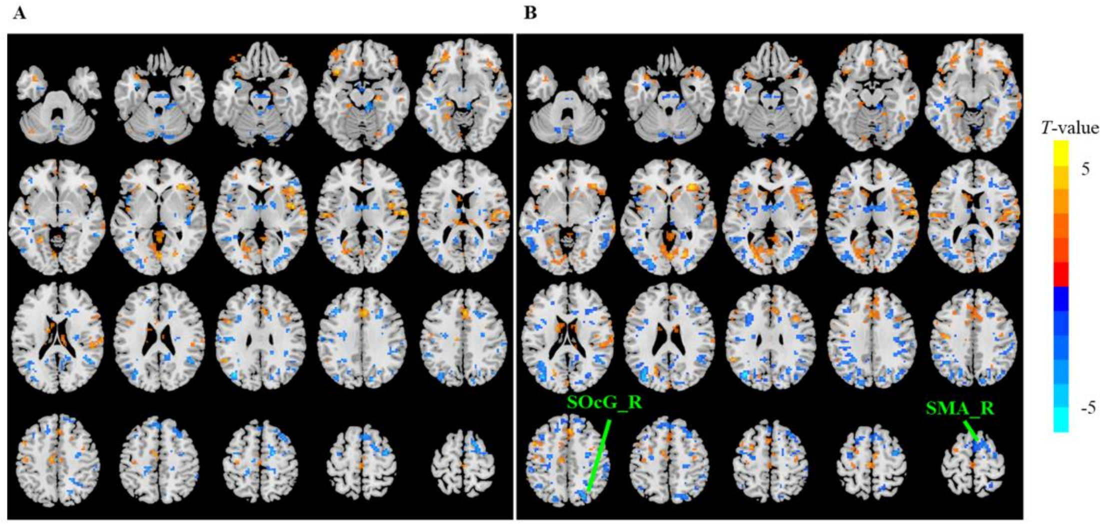

Supplementary Figure S7. The *t* map ($p < 0.05$, cluster size ≥ 10 voxels) of ADHD vs. TDC of NYU cohort in Slow-2 (0.1992 - 0.25 Hz) by FFT-ALFF **(A)** and db2-ALFF **(B)**, respectively. Cold colors indicate lower ALFF in ADHD. SOcG_R, right superior occipital gyrus; SMA_R, right supplementary motor area.

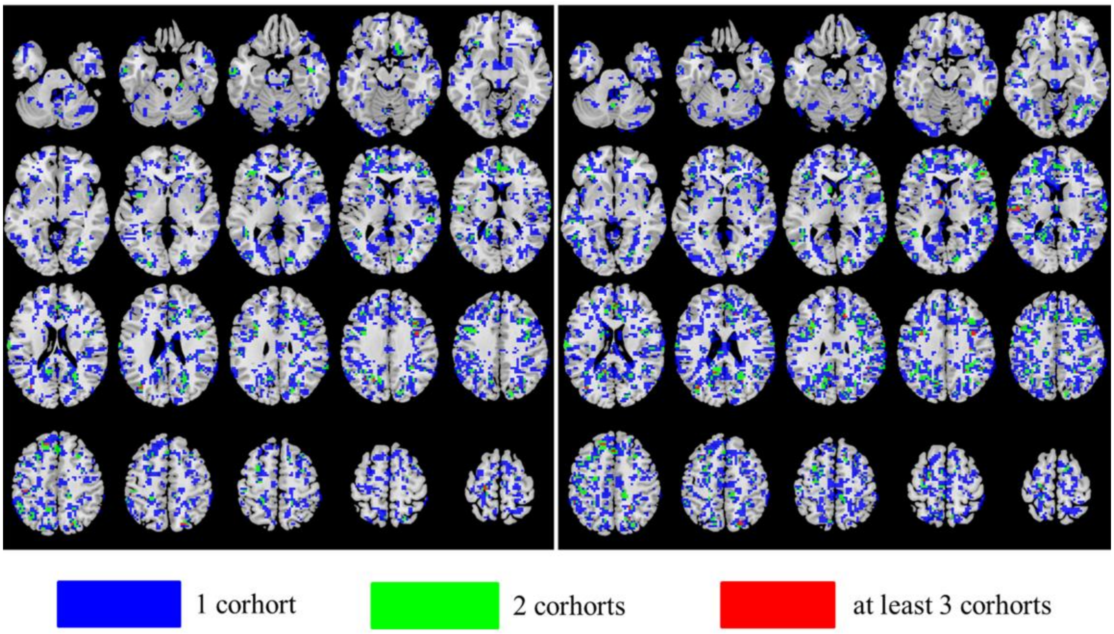

Supplementary Figure S8. The reproducibility brain maps across the 4 cohorts in Slow-6 (0 - 0.0117 Hz) by FFT-ALFF **(A)** and db2-ALFF **(B)**, respectively. Blue indicates the regions detected in only one of the 4 cohorts. Green and red indicate the regions detected in 2 and at least 3 cohorts, respectively.

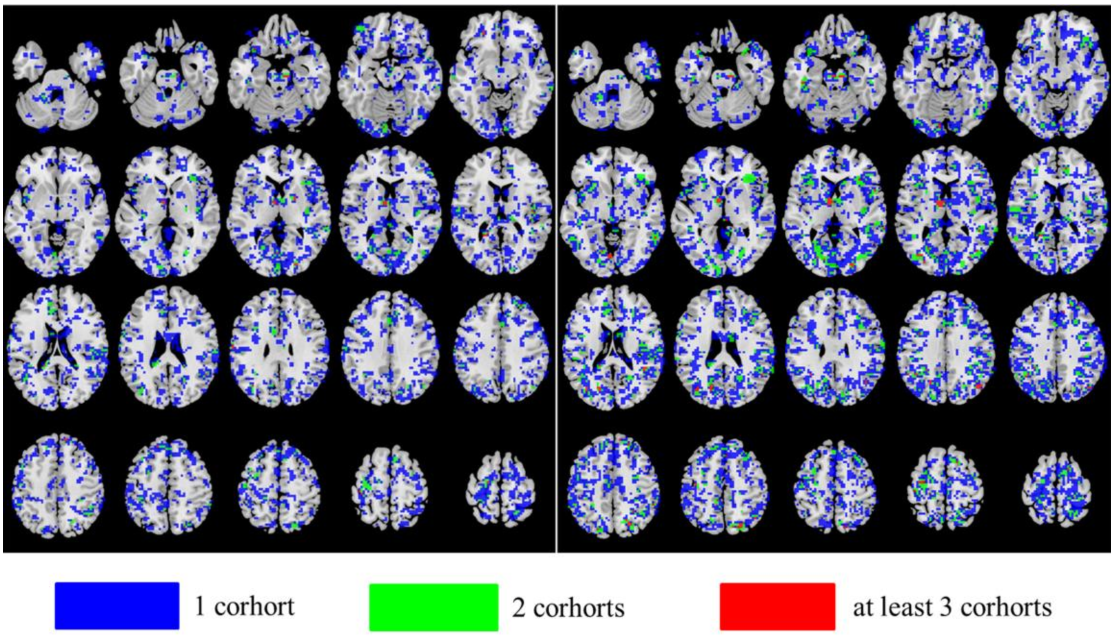

Supplementary Figure S9. The reproducibility brain maps across the 4 cohorts in Slow-2 (0.1992 - 0.25 Hz) by FFT-ALFF **(A)** and db2-ALFF **(B)**, respectively. Blue indicates the regions detected in only one of the 4 cohorts. Green and red indicate the regions detected in 2 and at least 3 cohorts, respectively.

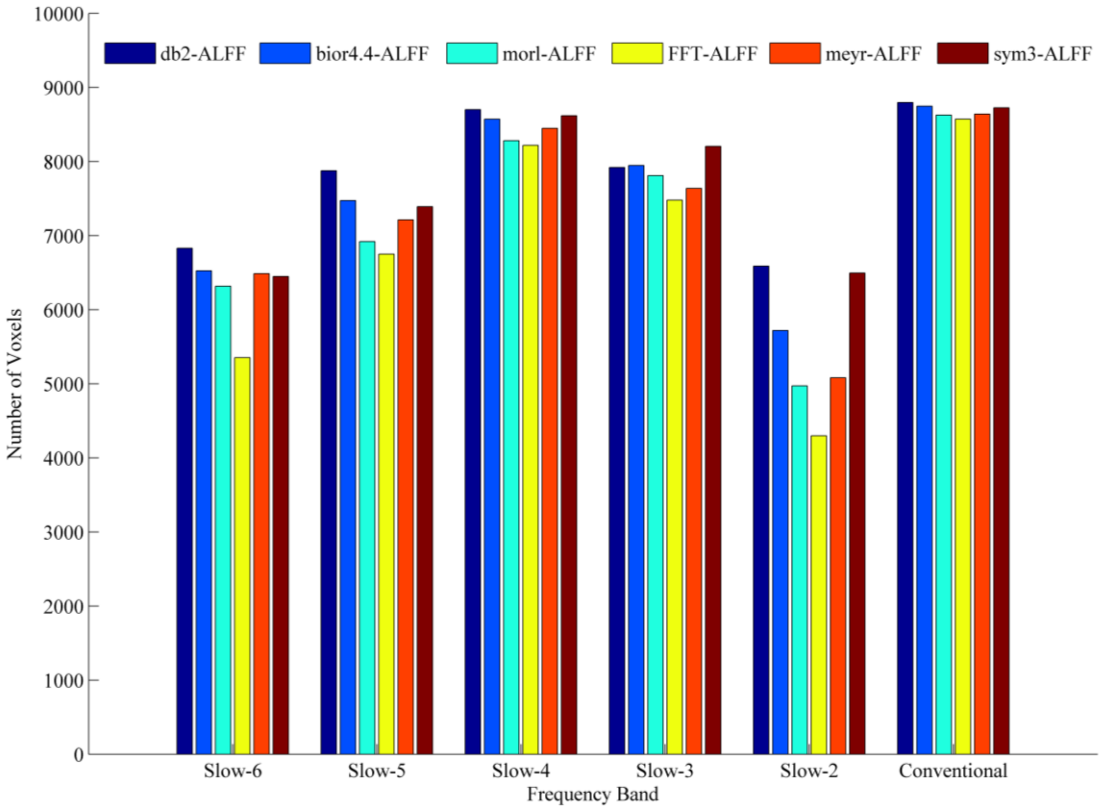

Supplementary Figure S10. The mean number of voxels showing significant difference in all 5 cohorts (NYU, PKU1, PKU2, PKU3, and EOEC)) calculated from Wavelet-ALFF and FFT-ALFF results in different frequency bands.
